# Maternity and Mothering

**Published:** 1911-10-14

**Authors:** 


### MATERNITY AND MOTHERING.

(Inquiries and Correspondence are invited)

### The Men and Women of To-morrow.

Since the administrative medicine of the future will more and more embrace within its purview the life of all citizens from the cradle to the grave, it is obvious that the many problems and perplexities which beset the nation's mothers and those who are concerned, privately or publicly, with their welfare demand the most careful attention from those whose outlook upon the national health is catholic rather than restricted. It has been said with some show of reason that this is the age of the child; and it is matter of rejoicing that it should be possible to say it, though much—very much—remains to be accomplished before our collective national treatment of childhood can be said to be adequate to our responsibilities in that respect. If the child is father of the man—and who will be hardy enough to deny that ancient saw?—even more is the infant the father of the child, and the unborn fœtus the grandfather of them all.

Granted these premises, and even in this age of scientific scepticism they seem to be unassailable, it follows that administrative medicine must consider well all that concerns the infant, born or unborn; and that involves the consideration also of the mother during the term of pregnancy. To go further back still is to encroach on the subject of eugenics, a topic which will one day have to be considered seriously by social reformers and public health administrators. That question is as old as Plato's day at least, and public sentiment is not yet educated up to its discussion in the spirit of sober earnestness which it demands and must eventually receive. It will, therefore, be excluded for our present purpose, which is to deal in these columns with all that concerns the mother and her family; their nature, upbringing, hygiene, and in fact all other matters pertaining to the welfare of the family in its early days. What the future of the family, regarded as an item of our established social system, is to be it would take a bold man to prophesy. But this much is certain, that the family has hitherto been the mainstay and the abiding *motif* of civilised society; and, Socialists notwithstanding, its dynasty still requires a great overthrow to dethrone it.

### Two Points of View.

Not only does this vastly important subject require attention from the institutional and administrative points of view, both of which are extensive enough, but even more is it to be regarded from the individual aspect. It is true enough that only some of us—even only some women—can ever expect to become actual mothers; but every woman has the chance at some time or other of "mothering" something or somebody, and it is not at all certain that all men are entirely devoid of the capacity and the aptitude for the same process. The chances may or may not be thrust upon one by relations or friends; but even if they are not they can in most cases easily be manufactured by a little trouble and a little thought. And thankless as the task of caring for a small child often appears, yet deep down in nature there is an instinct which only some such task can satisfy. Stifled and repressed as that instinct too often is by the artificial life of modern civilised society, it is none the less so deeply implanted that probably it can never be altogether extinguished. Indeed, were it otherwise the human race would long ago have ceased to exist.

If, then, it has been made plain that the problems of maternity, motherhood, and "mothering," so many of which are still unsolved, are problems which concern all citizens of the commonwealth, not only women but also men, it is clear that for institutional workers and for all concerned with the administrative as well as the practical side of medicine they must be full of interest. In these pages it shall be our aim to present aspects—some old, some new—of those problems from time to time as they appear in the light of modern research and modern theory. In the scope of the subject shall be included: from the individualistic side, the care of the expectant mother and the best ways of helping her before and during the birth of her offspring, the nursing at the puerperal stage, the proper methods of rearing infants, and all that concerns motherhood; from the social and administrative side, the institutions for receiving those whose circumstances make it impossible for them to be safely and suitably cared for at home, the associations whose work lies in superintending and supervising those who can and do stay at home, the economic aspect of work for new-made mothers and its influence upon the infant population of industrial communities, the midwife, her training and organisation, and many other kindred matters. Comment, inquiry, criticism will be at all times freely welcomed; advice always willingly tendered. The field is enormous, and there is room for many workers in it. Its exploration is a matter which touches vitally the future of the nation, and has already attracted a large number of enthusiastic workers. With yet more recruits, with enthusiasm, and with co-ordination, there is no doubt that much will be accomplished in a few years towards improving the quality of the nation's children, the men and women of to-morrow. It is doubtful if there is any other problem now urgently calling for solution which is so many-sided and so difficult as this. Essentially it is a problem of economics, of the regulation of social and political systems to the advantage of the mother, actual and prospective. For industrialism and poverty are the roots of the matter; too often medical science and hygiene can only shut the door after the horse has been stolen. Since causes which cannot at present be controlled are steadily diminishing our birth-rate, at least it must be seen to that our scanty children have the best possible chance to grow up strong and healthy; and to this end the scientific and practical study of maternity and mothering forms far and away the surest and quickest means.

